# The relationship between social roles and psychosocial adjustment in women with HIV: A structural equation model

**DOI:** 10.1002/brb3.1943

**Published:** 2020-11-20

**Authors:** Shadi Harifi, SeyedAhmad SeyedAlinaghi, Mostafa Qorbani, Zohreh Mahmoodi

**Affiliations:** ^1^ Student Research Committee Alborz University of Medical Sciences Karaj Iran; ^2^ Iranian Research Center for HIV/AIDS, Iranian Institute for Reduction of High Risk Behaviors Tehran University of Medical Sciences Tehran Iran; ^3^ Non‐Communicable Diseases Research Center Alborz University of Medical Sciences Karaj Iran; ^4^ Endocrinology and Metabolism Research Center, Endocrinology and Metabolism Clinical Sciences Institute Tehran University of Medical Sciences Tehran Iran; ^5^ Social Determinants of Health Research Center Alborz University of Medical Sciences Karaj Iran

**Keywords:** HIV, physical health, psychosocial adjustment, social roles, structural equation model

## Abstract

**Background:**

The human immunodeficiency virus is a unique social phenomenon that affects almost all the aspects of the social and economic life of individuals. The present study was conducted for the path analysis of the relationship between social roles and psychosocial adjustment in women with AIDS.

**Methods:**

This cross‐sectional study was conducted in 2019 on 240 women with HIV who referred to the Counseling Center for Behavioral Diseases of Imam Khomeini Hospital in Tehran. The eligible individuals entered the study through convenience sampling, and data were collected using five questionnaires, including the Psychosocial Adjustment to Illness Scale, the Perceived Social Support Scale, the Multidimensional Health Locus of Control Scales, the Illness Perception Questionnaire, the Experiences in Close Relationship Scale, and a socio‐demographic checklist. Data were analyzed using SPSS 16 and Lisrel 8.8 software.

**Results:**

According to the results of the path analysis, internal health locus of control and patient's age have a significant positive relationship with psychosocial adjustment only through the direct path, and among them, patient's age has the greatest positive and direct relationship (B = 0.139). In the indirect path, education, external health locus of control, anxiety in experiences of close relationships, and perceived support have the greatest relationship with psychosocial adjustment, and among these variables, education has the highest positive relationship (B = 0.06). Illness perception, avoidance in experiences of close relationships, and chance locus of health control are associated with psychosocial adjustment through both direct and indirect paths, and among these variables, chance locus of health control has the greatest overall relationship with psychosocial adjustment (B = 0.238).

**Conclusion:**

Based on the results, social roles and physical health are directly/indirectly associated with psychosocial adjustment, which emphasizes the need to pay more attention to all three aspects of psychosocial health, especially in this vulnerable group of the society.

## Graphical abstract

Patients with HIV are faced with numerous physical and mental health problems. The WHO offered a three‐dimensional definition of health encompassing biological, psychological, and social aspects. In most countries, however, both the psychological and social dimensions of health have been largely overlooked by health system administrators and country planners, and Iran has not been exempted from this rule either.

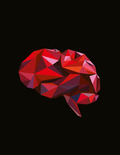

## 1 BACKGROUND

The human immunodeficiency virus (HIV) is not only one of the major challenges and health threats in the world, but is also now recognized as a biomedical and sociocultural issue (Behmanesh et al., 2016). According to World Health Organization's (WHO) reports, the number of women infected with the virus worldwide has reached more than 50% of all the cases. In Iran, the rate of HIV‐positive women has risen from 7 percent in 2015 to 11 percent in 2016 and 17 percent in 2017, which means both increasing cases of infection in women and also increased access to HIV testing among women (Gheiratmand et al., 2005).

This disease is a unique social phenomenon that affects almost every aspect of the social and economic life of individuals (Ahmadnia et al., 2018). It also leads to decreased self‐esteem and increased vulnerability, isolation, confused thoughts, and mental problems such as depression, anxiety, stress, and sleep disorders in patients (Allahyari & Eslamian, 2016).

Patients with HIV are faced with numerous physical and mental health problems. In 1946, the WHO offered a three‐dimensional definition of health encompassing biological, psychological, and social aspects. In most countries, however, both the psychological and social dimensions of health have been largely overlooked by health system administrators and country planners, and Iran has not been exempted from this rule either (noorbala, 2011).

Mental health means proper adjustment to oneself and the social environment. Proper adjustment allows the patients to make changes that guarantee their health and because of that the concept of 'Psychosocial Adjustment to Illness has received great attention over the past few decades (Samadzade et al., 2014). Adjustment refers to the personal characteristics that each person uses for their psychosocial management and to improve their life (Afrasiabifar et al., 2010). Psychosocial adjustment or adaptation refers to people's capacity to adapt to the environment, which implies that the individual has sufficient mechanisms to feel good, integrate, respond adequately to the demands of the environment, and achieve their objectives (Madariaga et al., 2014).

**FIGURE 1:**
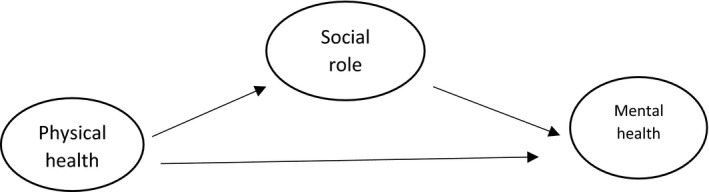
Social role quality and the mental health of women with chronic diseases (Plach et al., 2006). Black line: relation between variable. Black oval: study variable

Social roles are one of the most important factors affecting adjustment to illness. Based on the proposed model for the relationship between social role quality and mental health (Figure 1), women with chronic diseases who have a high social role quality adjust better to their disease (Plach et al., 2006). People play different social roles in the society, and these roles are the result of personal and group efforts by them to reap the benefits of their accumulated roles and prevent the incidence of social problems, such as the feeling of social alienation so it can assess by instruments that cover this concept. (Seyedan & Hesami, ).

According to this model, physical health is another factor that affects mental health and adjustment to illness. Physical health was assessed with four instruments that measured physical function, symptoms, health problems, and perception of health. Limitations in physical function were measured with a subscale of the Medical Outcomes Study‐HIV Health Survey (Plach et al., 2006). HIV symptoms and its health problems were assessed by a checklist, and perception of health was assessed by locus of health control and Illness Perception Questionnaire. Locus of health control is a concept in psychology based on which the patient's perceptions of health control have three distinct dimensions: internal of health control, powerful others of control, and chance health of control. Health control, in the hope and prevention of disease, involved in chronic diseases (Sahranavard et al., 2017).

In the majority of studies in this group, only one or two aspects of the health dimension have been examined, and there are no or very limited studies on all the three dimensions of health, that is, physical, psychological, and social dimensions. In light of these issues and given the importance of HIV, this study seeks to perform a path analysis of social roles in relation to psychosocial adjustment in women with HIV.

This study aimed to investigate the relationship between social roles and psychosocial adjustment in women with HIV: A structural equation model.

## 2 METHODS

### 2.1 Design

This cross‐sectional study was conducted in 2019 at the Counseling Center for Behavioral Diseases of Imam Khomeini Hospital in Tehran. This center serves as the main referral center for patients with AIDS and has the highest records of AIDS cases and was selected for this reason.

### 2.2 Study population

According to the study by Plach et al. (2006) and considering an alpha coefficient of 0.05 and beta coefficient of 0.2 and a correlation of 0.18 between social role quality and physical health and well‐being, the sample size was estimated as 240 according to the following formula. To increase the accuracy by 10% and consider potential sample loss, the sample size was increased to 250.

$$N={\left(\frac{Z_{\alpha }+Z_{\beta }}{C}\right)}^{2}+3.$$

$$C=\ln\left(\frac{1+r}{1‐r}\right).$$

Sampling took place through the convenience method until the determined sample size was reached.

### 2.3 Inclusion criteria

Inclusion criteria are being an Iranian woman aged 18–45 years with HIV positive and having health records in the select center, a CD4 count < 500 (as per the center's treatment protocol), minimum reading and writing literacy, a sexual partner, and they did not have any psychological or physical illnesses based on self‐report, medical file, or doctor's diagnosis.

### 2.4 Exclusion criteria

Exclusion criteria are incomplete completion of the questionnaires and the self‐reported use of psychotropic substances during the study or illicit drugs that could affect their answer.

### 2.5 Data collection and definition of terms

Data were collected using five questionnaires, including the Psychosocial Adjustment to Illness Scale (PAIS), the Perceived Social Support Scale (PSSS), the Multidimensional Health Locus of Control Scales, the Illness Perception Questionnaire (IPQ), the Experiences in Close Relationships (ECR) Scale, and a socio‐demographic checklist.

To examine social roles, which are defined as the product of personal and group efforts by individuals to reap the benefits of their accumulated roles and prevent many social problems, such as the feeling of social alienation (Sieber, 1974), three questionnaires were used, namely the ECR, PSSS, and a socio‐demographic checklist. In this study, physical health was assessed using two questionnaires, namely the Multidimensional Health Locus of Control Scales and the IPQ.

### 2.6 Socio‐demographic checklist

A researcher‐made checklist including items on age, education, occupation, marital status, insurance status, number of children, and method of affliction was completed for all the participants.

### 2.7 'Psychosocial Adjustment to Illness scale

The Psychosocial Adjustment to Illness Scale (PAIS) consists of 46 items in seven domains, including health care orientation, vocational environment, domestic environment, sexual relationships, extended family relationships, social environment, and psychological distress. Scoring is on a 4‐point scale from zero to three. The sum of the scores of each component was divided by the number of items in that component and the mean was considered that component's compatibility score, and the sum of all the scores was divided by the total number of items and the mean total score was taken as the total score. The validity and reliability of this tool were calculated in Iran in 2013, and the scale's Cronbach's alpha was calculated as 0.94 (Derogatis, 1992). The present study assessed the reliability of the tool in the target group, and Cronbach's alpha coefficient for the total score of psychosocial adjustment was calculated as 64.1.

### 2.8 Experiences in close relationships (ECR) scale

The Experiences in Close Relationships (ECR) is a 36‐item tool that measures adult attachment in two dimensions (18 items each), namely attachment‐related anxiety and attachment‐related avoidance in close relationships. The items are answered on a Likert scale from “strongly agree” to “strongly disagree” (1–7), and each person receives a score from 6 to 42 in the avoidance dimension and 3 to 21 in the anxiety dimension of each domain of relationship. The first four items are reverse scored (Brennan et al., 1998). The validity and reliability of this questionnaire have been assessed in Iran by Panaghi et al. in 2014 (Panaghi et al., 2014). The present study also assessed the reliability of this questionnaire in the target group, and its Cronbach's alpha coefficient was found as 63.3% for anxiety and 84% for avoidance.

### 2.9 Perceived social support scale

The Perceived Social Support Scale (PSSS) is a 12‐item tool designed by Zimet et al. (1988) to assess perceived social support from three sources, namely the family, friends, and significant others. To obtain the total score, the scores of all the scale items are summed up and divided by their number –that is, 12 (Zimet et al., 1988). The present study assessed the reliability of the questionnaire in the target group, and the Cronbach's alpha for social support was reported as 0.91.

### 2.10 Illness perception questionnaire

The Illness Perception Questionnaire (IPQ) was designed by Broadbent et al. in nine subscales based on the revised form of this questionnaire, and its first eight items are scored from 1 to 10 while item nine is open‐ended and asks about the three major causes of the disease in respective order. Each subscale measures one component of illness perception. In Iran, the validity and reliability of this questionnaire were examined by Bazzazian. Cronbach's alpha coefficient of the questionnaire was reported as 0.8, and its retest reliability coefficient after a 6‐week interval was reported as 0.42 to 0.75 for the different items (Broadbent et al., 2006). The present study examined the reliability of this questionnaire in the target group and reported Cronbach's alpha coefficient of 62.4 for the entire scale.

### 2.11 Multidimensional health locus of control scales

The Multidimensional Health Locus of Control Scales was developed by Wallston and DeVellis in 1978 (Wallston et al., 1987). This questionnaire has 18 items in three subscales (internal of health control, powerful others of control, and chance health of control). Each scale consists of six items, which are measured using a 6‐point Likert scale (“strongly disagree” = 1 point to “strongly agree” = 6 points). In Iran, this scale was first translated and localized by Meshki, Ghafrani Pour, Azad Fallah, and Hajizadeh (2007), and the reported coefficients were 70% for internal health locus of control, 75% for powerful others locus of control, and 69% for chance locus of control (Moshki et al., 2007). The present study re‐evaluated the reliability of each dimension in the target group and reported Cronbach's alpha coefficients of 47.5 for the internal locus, 59.2 for the powerful others locus, and 58.6 for the chance locus.

### 2.12 Procedures

An ethics code was obtained from the Ethics Committee of Alborz University of Medical Sciences (ABZUMS.REC.1397.195) and the Ethics Committee of the Counseling Center for Behavioral Diseases of Imam Khomeini Hospital in Tehran (IR.TUMS.VCR.REC.1398.435).

First, the researcher visited the counseling center of Imam Khomeini Hospital and identified the eligible candidates and briefed them on the study objectives. Written consent was then obtained from them if they wished to participate in the study. The study questionnaires were then distributed among them to answer. The researcher was present at the center during this stage and answered any potential questions the respondents had. If the questionnaires could not be completed in a single session, the subjects were invited for a follow‐up visit. There was no obligation or imposition to complete the questionnaires in the first session, and they could be completed at any time during the research. A separate space was allocated in the center for this purpose, so that the questionnaires could be completed without stress and others’ presence.

### 2.13 Statistical analyses

This study examined the fit of a conceptual model of path analysis (Figure 1) to determine the concurrent relationship of psychosocial adjustment variables, social roles (experiences in close relationships, perceived support, and socio‐demographic characteristics), and physical health (locus of health control, illness perception). First, the normality of the quantitative variables was assessed by the Kolmogorov–Smirnov test. Path analysis is a generalization of the normal regression that, in addition to expressing the direct effects, also demonstrates the indirect effects and the effect of each variable on the dependent variables and can be used to provide a rational interpretation of the observed relationships and correlations (Vieira, 2011). Data were analyzed in Lisrel 8.8 and SPSS 16 software. The correlation results were analyzed using Pearson's correlation coefficient, and the path analysis was expressed as beta. The level of significance was set at T‐values > 1.96.

## 3 RESULTS

In this study, data were collected from 240 women with acquired immunodeficiency syndrome/AIDS. According to the findings, the mean age of the participants was 36.7 ± 6.6 years and the duration of their relationship with their sexual partner was 10.7 ± 4.4 years, and 88.3% of the subjects had a sexual partner (Table 1). According to the results of Pearson's correlation test, psychosocial adjustment was significantly correlated with internal and external loci of health control health control and chance, illness perception, perceived support, anxiety and avoidance in close relationships, and patient's education. Among these variables, illness perception had the most significant positive correlation with psychosocial adjustment (*r* = .239, *p* = .01) (Table 2).

**Table 1:** Socio‐demographic characteristics of participants

Variables(quantitative)			Mean ± *SD*	Minimum	Maximum
Age (year)			36.7 ± 6.6	18	49
Relationship duration with their sexual partner (year)			10.7 ± 6.4	1	35
Duration of disease diagnosis (year)			5.4 ± 4	1	30
Variables (qualitative)			*F* (%)	Variables	*F* (%)
Number of sexual partners	<2	213 (88.7)	Education	Illiterate	30 (12.5)
	≥2	27 (11.3)		Elementary and middle school	113 (47.1)
Number of children	Zero	51 (21.3)		High school and diploma	89 (37.1)
	1	72 (30)		BS & MS	8 (3.3)
	2	56 (23.3)			
	3 and more	61 (25.4)			

**Table 2:** Correlation between health locus of control, illness perception, social roles, and Psychosocial Adjustment to Illness in women with HIV

Variable	PAD	ANCR	AVCR	PSS	IP	CHLC	PHLC	IHLC	RDS	HIVD	CHN	EDU	AGE
AGE	0.106	−0.083	0.11	0.058	0.052	0.033	−0.73	−0.018	0.483**	0.078	0.298**	0.068	1
EDU	0.175**	0.149	0.176**	−0.130*	0.241**	−0.049	−0.034	0.04	0.03	−0.55	−0.181**	1	
CHN	−0.79	−0.130*	−0.123	0.182**	0.078	0.065	0.006	0.064	0.367**	−0.139*	1		
HIVD	0.101	0.008	0.085	−0.061	0.109	−0.03	0.058	−0.012	0.065	1			
RDS	0.011	0.024	−0.086	0.16	0.012	0.033	−0.049	−0.034	1				
IHLC	0.219**	−0.205**	−0.174**	0.134*	0.175**	0.557**	0.516**	1					
PHLC	0.144*	−0.135*	0.151*	0.025	0.008	0.428**	1						
CHLC	0.188**	−0.150**	−0.229**	0.211**	0.007	1							
IP	0.239**	0.096	0.061	0.115	1								
PSS	−0.07**	−0.265**	−0.55**	1									
AVCR	0.200**	0.367**	1										
ANCR	0.130*	1											
PAD	1												

Abbreviations: AGE, age of women with HIV; ANCR, attachment‐related anxiety in close relationships; AVCR, attachment‐related avoidance in close relationships; CHLC, chance health locus of control; CHN, child number; EDU, education years; HIVD, duration of disease diagnosis; IHLC, internal locus of health control; IP, illness perception; PAD, Psychosocial Adjustment to Illness; PHLC, powerful others locus of control; PSS, total perceived social support; RDS, relationship duration with their sexual partner (year).

** Correlation is significant at the 0.01 level (2‐tailed).

* Correlation is significant at the 0.05 level (2‐tailed).

**FIGURE 2:**
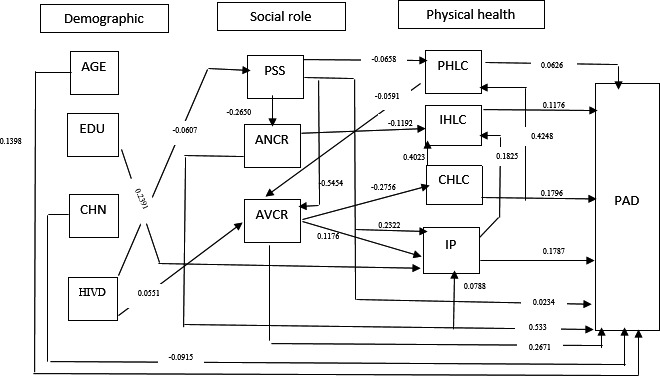
Full Empirical Model (Empirical Path Model between Health Locus of Control, Illness Perception, social roles and Psychosocial Adjustment to Illness in women with HIV) RDS, Relationship Duration with their sexual partner (year), PSS, total Perceived social support IP, Illness Perception, CHLC, chance health locus of control, PHLC, powerful others locus of control, IHLC, Internal locus of health control, ANCR, attachment‐related anxiety in close relationships, AVCR, attachment‐related avoidance in close relationships, PAD, Psychosocial Adjustment to Illness, CHN, child number EDU, education years, HIVD, Duration of disease diagnosis, AGE, age of women with HIV

Based on the results of the path analysis (Figure 2), the internal locus of health control and patient's age were variables that were positively and significantly correlated with psychosocial adjustment through only one path, which is direct, and patient's age had the highest positive direct correlation among them (B = 0.13). In other words, patients’ psychosocial adjustment increases with age. The next variable was the internal locus of health control, which had a significant, direct, and positive correlation with psychosocial adjustment (B = 0.11). This variable indicates the person's belief in internal factors and own behaviors being responsible for his illness and health, and the higher are these beliefs, the higher becomes the individual's psychosocial adjustment.

According to the findings, education, external locus of health control, anxiety in experiences of close relationships, and perceived support are significantly related to psychosocial adjustment only through one path, which is indirect. Among these variables, education has the highest positive relation with psychosocial adjustment through illness perception (B = 0.05). In other words, the higher is the individual's education, the higher becomes their social–psychological adjustment. Perceived support was the second variable that had the highest significant positive relation with psychosocial adjustment through the indirect path (B = 0.04). In other words, higher perceived support by the individual is associated with increased psychosocial adjustment through the mediating variables. Illness perception, avoidance in experiences of close relationships, and chance locus of health control are related to psychosocial adjustment through both direct and indirect paths. Among these variables, the chance locus of health control had the highest general relationship with psychosocial adjustment (B = 0.23). This variable indicates the individual's degree of belief in his health depending on chance, fortune, fate and destiny, and the higher it is, the higher gets the degree of psychosocial adjustment. After this variable, illness perception had the most significant positive relationship with psychosocial adjustment (B = 0.18). In other words, the more is the person's perception of illness, the higher is his psychosocial adjustment (Table 3).

**Table 3:** Path coefficients for Psychosocial Adjustment to Illness in women with HIV

Variable	Direct effect	Indirect effect	Total
Age	0.139^*^	–	0.139^*^
Education	–	0.0588^*^	0.0588^*^
Child number	–	–	‐
Duration of disease diagnosis	–	–	‐
Internal locus of health control	0.11^*^	–	0.11^*^
Powerful others locus of control	–	0.0363^*^	0.0363^*^
Chance health locus of control	0.179^*^	0.059^*^	0.238^*^
Illness Perception	0.17^*^	0.0198^*^	0.189^*^
Perceive social support	–	0.0484^*^	0.0484^*^
Attachment‐related anxiety in close relationships	–	−0.0131^*^	−0.0131^*^
Attachment‐related avoidance in close relationships	0.26^*^	−0.081^*^	0.179^*^

* indicates significant.

The results of the model fit indices show the favorableness and high fit of the model and the rationality of the regulated relationships between the variables based on the conceptual model. Accordingly, the fitted model is not significantly different from the conceptual model (Table 4).

**Table 4:** Goodness‐of‐fit indices for the model

Fitting Index	*X* ^2^	*df*	*X* ^2^/*df*	CFI	GFI	NFI	RMSEA
Model Index	60.26	35	1.72	0.96	0.97	0.92	0.055
Acceptable Range	*X* ^2^/*df* < 5	>0.9			>0.9	>0.9	<0.05

Abbreviations: GFI, goodness‐of‐fit statistic; NFI, normed‐fit index; RMSEA, root‐mean‐square error of approximation, ×2 chi‐square.

## 4 DISCUSSION

Based on the results of the structural equation model, psychosocial adjustment was significantly correlated with the internal locus of health control and age through the direct path and with education and perceived support through the indirect path. In addition, there was a significant correlation between psychosocial adjustment and illness perception, avoidance in close relationships, and locus of health control/chance through both direct and indirect paths.

As stated, age had the most significant correlation with psychosocial adjustment. This finding is in agreement with the results reported by Nathalie Hauk et al. in 2019, who found that, as employees’ age, they gain more adaptive skills and adjustment, which help reduce the negative consequences of stress (Hauk et al., 2019). According to psychological theories, aging is associated with greater flexibility, coping strategies, and competence in handling emotions (Diehl & Hay, 2010).

Internal locus of health control was another variable that was directly correlated with psychosocial adjustment. Health control plays a role in the hopefulness and prevention of illness in people with chronic diseases. This factor makes the person feel in control and plays a role in their stress reduction and better adjustment. People who believe that they can play a role in controlling and preventing their illness believe in behaviors such as praying, worshipping, lifestyle changes, and social interactions to improve their quality of life (Sahranavard et al., 2017). This finding is in line with the results reported by ZarrinPour and Aflakseir, who found that people who scored higher on this locus were more likely to adhere to their treatment regimens (Aflakseir & ZarrinPour, 2013).

Among the variables that were indirectly correlated with social psychosocial adjustment, education had the most significant positive correlation. In other words, psychosocial adjustment increases with the level of education. This factor contributed to this relationship through illness perception. Higher levels of education in patients with AIDS lead to improved knowledge and awareness about the disease (Faust et al., 2018). Mirowsky (2017) found that when people are more educated, their interpersonal relationships become more stable and they adjust better to the environment, which are crucial for adhering to treatments and the maintenance and promotion of their health. Brunello et al. also found that the higher are people's awareness and education, the less stress and better adjustment will they experience (Brunello et al., 2016).

Perceived support was the second variable that was most positively and significantly related to psychosocial adjustment on the indirect path. In other words, increased perceived support is associated with increased psychosocial adjustment. Shishegar et al. (2013) found that social support is indirectly correlated with quality of life in pregnant women (Shishehgar et al., 2013). In another study, Ahmadi et al. found that social support is a key determinant of health throughout life (Ahmadi, 2016). Koetsenruijter et al. reported that people with better social support have higher self‐esteem, which contributes to their health as well (Koetsenruijter et al., 2015).

Among the variables that were associated with psychosocial adjustment through both paths, the chance locus of health control showed the highest overall correlation. This variable indicates the degree to which the person believes in his health depending on chance, fate, fortune, and destiny, and the higher it is, the higher is the level of psychosocial adjustment. Neipp et al. found that control beliefs predict psychosocial adjustment in women with cancer (del Carmen Neipp et al., 2009). Illness perception was the second variable that was most correlated with psychosocial adjustment through both paths. Illness perception indicates the patient's organized cognitive representation of his illness and his beliefs about the different aspects of his new condition (Kalantari et al., 2012). Many studies have been conducted on illness perception. Barbasioa et al. concluded that the greater is the perception of illness in people with systemic lupus erythematosus, the less are they likely to develop disorders such as depression (Barbasio et al., 2015). Greco et al. also reported a significant correlation between illness perception and cardiovascular disease complications; that is, the lower was illness perception, the greater were cardiovascular disease complications (Greco et al., 2015). Zelber‐Sagi et al. also found a significant correlation between healthy eating habits and healthy lifestyle in patients with fatty liver (Zelber‐Sagi et al., 2017).

## 5 CONCLUSIONS

Based on the results, social roles (perceived social support, experiences in close relationships, and perceived support), and physical health (health locus of control and illness perception) are either directly or indirectly or else through both paths correlated with psychosocial adjustment, which emphasizes the importance of paying greater attention to all three aspects of health, that is, physical, psychological, and social aspects, especially in this vulnerable group of the society.

### 5.1 Study limitations

This study was conducted only on women with HIV positive and their spouses or sexual partners were not included due to their limited accessibility, which comprises one of the limitations.

## CONFLICT OF INTEREST

The authors declare that they have no competing interests.

## AUTHOR CONTRIBUTIONS

SH collected the data and statistical analysis and wrote the manuscript.ZM has supervised the study, contributed to the study design, conducted the analysis, and wrote the manuscript. MQ analyzed the data and draft the manuscript. SASA helped as scientific adviser. The author(s) read and approved the final manuscript.

## ETHICS APPROVAL AND CONSENT TO PARTICIPATE

Informed consent was obtained from all individual participants included in the study. An ethics code was obtained from the Ethics Committee of Alborz University of Medical Sciences (ABZUMS.REC.1397.195) and the Ethics Committee of the Counseling Center for Behavioral Diseases of Imam Khomeini Hospital in Tehran (IR.TUMS.VCR.REC.1398.435).

### Peer Review

The peer review history for this article is available at https://publons.com/publon/10.1002/brb3.1943.

## Data Availability

The data that support the findings of this study are available from the corresponding author upon reasonable request.
